# National Service Scheme (NSS) Training in dental education: Assessment of self reported empathy and clinical performance

**DOI:** 10.4317/jced.62130

**Published:** 2024-10-01

**Authors:** Gadde Praveen, Villuri Sherly PrabhaLatha, Pasupuleti Mohan Kumar, Anitha Akkaloori, K Swathi, Koothati Ramesh Kumar

**Affiliations:** 1 Associate Professor, Department of Public Health Dentistry, Vishnu Dental College, Bhimavaram, India; 2 Undergraduate, Department of Public Health Dentistry, Vishnu Dental College, Bhimavaram, India; 3 Associate Professor, Department of Periodontics, Vishnu Dental College, Bhimavaram, India; 4 Assistant Professor, Department of Public Health Dentistry, Government Dental College & Hospital, Hyderabad, Telangana, India; 5 Professor, Department of Public Health Dentistry, Malla Reddy Institute of Dental Sciences, Hyderabad, Telangana, India; 6 Professor & HOD, Dept Of Oral Medicine & Radiology, Government Dental College & Hospital, Vijayawada, Andhra Pradesh, India

## Abstract

**Background:**

To compare self-reported empathy scores, clinical performance between National Service Scheme (NSS) volunteers and non-volunteers in dental schools; and to predict clinical performance score using self-reported empathy score.

**Material and Methods:**

A cross sectional survey of 336 undergraduates from 16 dental schools in Andhra Pradesh state, India was conducted using Google forms. The questionnaire was divided into four sections. Section A consists of a single question asking whether a graduate is a NSS volunteer or not. Section B consisted of Jefferson Scale of Empathy Health Profession Students’ Version (JSPE-HPS) with 20 items based on a 7-point Likert scale. Section C was designed to investigate students’ self-confidence in performing 35 clinical procedures on a 5-point Likert scale. Section D gathered information related to clinical exam score in external practical assessment during final year. The data collected were subjected to appropriate statistical tests.

**Results:**

The JSPE-HPS score between NSS volunteers (91.52+7.35) and non-volunteers (76.21+5.42) was significantly different. The overall self-reported clinical score was 3.57+.34 and 3.08+.43 for NSS volunteers and non-volunteers respectively (*p*=0.000). Also, the mean clinical performance score was higher for NSS volunteers (501.15+53.97) compared to non-volunteers (445.03+34.94). The JSPE-HPS scoreswere positively associated with clinical performance scores (r=0.559). Furthermore, we discovered that JSPE-HPS scoresignificantly predicted clinical exam score (β = 2.959, *p*< .000).

**Conclusions:**

NSS training enabled dental undergraduate students acquire empathy and clinical skills during their education.

** Key words:**Clinical Skills, Dental Education, Empathy, National Service SchemeCare Team.

## Introduction

The National Service Scheme (NSS) is a Government of India sponsored community service program under the ministry of Youth Affairs and Sports (1,2). The basic rule of the NSS is that it is structured by the students with the objective of developing their personality through community service (1). All dental schools in India have a NSS unit and each unit has a minimum of 100 dental undergraduates as volunteers. The volunteers spend a minimum of 240 hours of work related to NSS during their academic career after which they receive a certificate of appreciation (3). NSS volunteers have the chance to become involved in society through community service. It helps volunteers to think critically, solve problems, and make decisions in situations that are personally relevant to them. Along with traditional classroom learning, it promotes social responsibility, cooperation, and tolerance in students. It establishes connections between theory and practice, between knowledge and action, and between community and institutional resources (1-3). The community service done under NSS by volunteers also aid in developing empathy (3).

Empathy is a key component of dentist patient relationship as it enables them to identify and understand patient’s experiences, concerns, and perspectives (4).Empathy among dental graduates is said to improve patient outcomes by lowering anxiety and stress, boosting patient satisfaction, and promoting treatment compliance. Additionally, it has been noted that specific aspects of empathetic behaviours, such as eliciting information regarding psychosocial concerns, socioemotional behaviours, problem-solving techniques, and emotion-management abilities, are favourably associated with patient outcomes (5,6). An empathic health‑care provider is happier at work, enjoys patient visits, and is clinically competent (5).

There is unquestionable importance of empathy in dental education (7). Many studies have been conducted to assess empathy levels among dental students (5,7-20). However, lacunae exist in research to understand how NSS influence empathy among dental students. Also, studies examining the correlation between empathy levels and clinical performance are limited. Hence, this study was conducted to assess and compare self-reported empathy score andself-confidence in performing various clinical procedures between NSS volunteers and non-volunteers; and to correlate self-reported empathy with the clinical exam scoreduring final year of graduation.

## Material and Methods

Institutional Ethics Committee approved the study protocol. The study included dental graduates from 16 dental schools in Andhra Pradesh state, India. The graduates who were the part of NSS unit of their respective dental school at least for 2 years were considered as NSS volunteers and the remaining as non-volunteers.

An online survey using Google forms was conducted. The survey was dispatched to all 1055 graduates who had completed their final year during the academic year 2022–2023; hence sample size estimation was not done. Initial emails were sent on April 14, 2023, followed by reminder emails after 15 days and 1 month. The responses were collected on May 15, 2023. Online consent was required for participation, and graduates who declined were not included in the study. No personal information was gathered from the study participants.

The questionnaire was designed by two authors. A third opinion was sought in case of disagreement. The questionnaire was divided into four sections. Section Aconsists of a single question asking whether a graduate is a NSS volunteer or not. Section B consisted ofJefferson Scale of Empathy Health Profession Students’ Version (JSE-HPS) (21). The JSE-HPS instrument contained 20 items with response options based on a 7-point Likert scale (strongly disagree=1, strongly agree=7). The 10 negatively worded items in the scale were reverse scored. A higher score shows a more empathic orientation toward patient care. The clinical performance of graduates was assessed in two ways. In section C, graduates were asked to self-rate their skills in performing 35 clinical procedures using a 5-point Likert scale (very poor=1, very good=5). The Dental Council of India outlined these 35 procedures as being necessary for students to perform after completing their final year (22). In section D, the clinical performance was assessed by asking them to self-report the cumulative clinical exam score provided by external examiners during the final BDS for eight specialties. The highest possible score was 800 (100 for each specialty). A pilot survey was done on 50 students to validate the questionnaire. Cronbach’s alpha value of 0.891 indicates an excellent internal consistency.Test-retest reliability coefficients were good at 0.802.

Data were statistically analysed using SPSS software version 25.0 (IBM Corp, Armonk, NY, USA). Descriptive analyses were done. The Mann-Whitney U test was performed to compare empathy levels, clinical performance scores between NSS and Non NSS volunteers. Spearman’s test was used to determine the correlations. Simple linear regression was used to predict clinical exam score using self-reported empathy. Statistical significance was set at *p* <0.05.

## Results

In total, 336 graduates from 16 dental schools in Andhra Pradesh state responded to the survey. The response rate was 31.8%. Of these, 180 (53.57%) were Non volunteers and 156 (46.42%) were NSS volunteers.  Table 1 shows the comparison of mean empathy levels between the groups. The mean score was higher for NSS volunteers compared to non-volunteers for all the items of empathy scale except for item 2 (*p*=323). There was also a significant difference in overall empathy score between NSS volunteers (91.52+7.35) and non-volunteers (76.21+5.42). (*P*=0.000).

 Table 2 presents the self-reported clinical scores for 35 items in both the groups. There was a significant difference in self-reported clinical scores between the groups for all the items except for item 5,11,18, 20, 22, 24, 33 and 34 (*p*>0.05). The overall self-reported clinical score was 3.57+.34 and 3.08+.43 for NSS volunteers and non-volunteers respectively (*p*=0.000). Also, the mean practical exam score was higher for NSS volunteers (501.15+53.97) compared to non-volunteers (445.03+34.94) and the difference was statistically significant (*p*=0.000).

A Pearson correlation coefficient was computed to assess the linear relationship between empathy levels and clinical exam score. There was a positive correlation between the two variables, r (334)=.559, *p*=.000.Simple linear regression was used to test if empathy levels significantly predicted clinical exam score. The fitted regression model was: Clinical exam score=224.51+2.959*(empathy score). The overall regression was statistically significant (R2 =.312, F(1, 334)=151.496, *p* < .000). It was found that empathy score significantly predicted clinical exam score (β = 2.959, *p* < .000).

## Discussion

We demonstrated that the NSS training enabled dental undergraduate students acquire empathy during their education. Dental education in India has a special emphasis on NSS. The dental graduates as NSS volunteers involved in various community services like adoption of villages for oral health promotion, carrying out oral health surveys, setting up of dental screening and treatment centres, oral health education programmes etc. Since no study has evaluated empathy levels of NSS volunteers in dental schools, we made a comparison with other disciplines. In a study conducted by KhandareKiran and DesaiPriti (1) it was found that NSS helped undergraduate students of Ayurveda in developing communication skill and leadership. In another study by Dharmayat S (3) it was demonstrated that Physiotherapy students who were part of the NSS team developed team building, leadership qualities and life-skills. Biswamitra Purohit and Iswar Patel (2) demonstrated that NSS programme is a platform to develop emotional intelligence among volunteers. From the above it is evident that NSS training helps students to develop multi dimensionally during their career in respective field.

We also showed that self confidence in performing various clinical procedures and practical exam scores were higher for NSS volunteers compared to Non volunteers. Also, Self-reported empathy scores were positively associated with the practical exam scores. In a similar study conducted by Casas RS *et al*. (23) it was found that empathy levels among medical students at Boston University School of Medicine were positively associated with clinical competence. In another study by Watanabe S *et al*. (5) a positive relationship was established between trainee dentists’ self-reported empathy and communication behaviours with simulated patients’ assessment in medical interviews. It is clearly evident from the findings thatself-reported empathy could predict clinical performance.

Contrary to this, self-reported empathy was not linked to clinical performance among Australian medical students (24). Another British study found that empathy scores were not linked to indicators of all-around academic success among medical students at Edinburg university (25). These variations in correlations between empathy and clinical skills may be influenced by various grading and training frameworks. Further studies are needed to ascertain the potential discrepancy between self-reported and observed empathy scores and more comparisons of self, faculty, standardized patient, and real patient-reported empathy measures are required.Although self- and observer ratings vary, the patient will always need an empathic dentist, thus graduates should be prepared with the requisite abilities to be empathetic to the patient (24).

Future research should consider the strengths and limitations. The study included graduates from all dental schools in Andhra Pradesh state with a good response rate. It is not considered to be reliable when empathy, clinical performance scores are self-reported. Hence, a study with more objective assessment methods could provide a better direction for future research.

## Conclusions

The dental undergraduates as NSS volunteers had reported better empathy scores and clinical skills compared to non-volunteers. The self-reported empathy scores were positively associated with clinical exam score and found to be predictive on regression analysis. These findings add to the body of literature indicating that enrolling graduates under NSS and promoting an empathic attitude is an important aspect in the dental education.

## Figures and Tables

**Table 1:** Comparison of self-reportedempathy scores between NSS and Non NSS volunteers.

Items	Non NSS volunteers		NSS volunteers		P value
	Mean	SD	Mean	SD	
Health care providers' understanding of their patients' feelings and the feelings of their patients' families do not influence treatment outcomes.	3.528	1.1058	4.571	1.0103	0.000*
Patients feel better when their health care provider understands their feelings.	4.450	.8540	4.564	1.2453	0.323
It is difficult for a health care provider to view things from patients' perspectives.	2.978	.9333	4.000	1.2649	0.000*
Understanding body language is as important as verbal communication in health care provider – patient relationships.	4.456	1.0848	5.000	1.3001	0.000*
A health care provider's sense of humor contributes to a better clinical outcome.	4.200	1.0270	4.712	1.3959	0.000*
Because people are different‚ it is difficult to see things from patients' perspectives.	2.922	1.0435	4.109	1.0569	0.000*
Attention to patients' emotions is not important in patient interview [interviewing].	3.483	1.4892	4.615	.7136	0.000*
Attentiveness to patients' personal experiences does not influence treatment outcomes.	3.589	1.1126	4.353	.7432	0.000*
Health care providers should try to stand in their patients' shoes when providing care to them.	4.294	.9136	4.872	1.3379	0.000*
Patients value a health care provider's understanding of their feelings‚ which is therapeutic in its own right.	4.306	1.0089	4.814	1.2690	0.000*
Patients' illnesses can be cured only by targeted treatment; therefore‚ health care providers' emotional ties with their patients do not have a significant influence in treatment outcomes.	3.444	1.1395	3.910	.9324	0.000*
Asking patients about what is happening in their personal lives is not helpful in understanding their physical complaints.	3.206	1.2627	3.981	.5503	0.000*
Health care providers should try to understand what is going on in their patients' minds by paying attention to their non-verbal cues and body language.	4.244	1.0915	4.519	1.2416	0.032*
I believe that emotion has no place in the treatment of medical illness.	3.150	1.0907	4.346	1.2631	0.000*
Empathy is a therapeutic skill without which a health care provider's success is limited.	4.317	1.1505	5.103	1.3060	0.000*
Health care providers' understanding of the emotional status of their patients‚ as well as that of their families is one important component of the health care provider – patient relationship.	4.433	.9160	5.199	1.2619	0.000*
Health care providers should try to think like their patients in order to render better care.	4.072	1.2283	4.968	1.4342	0.000*
Health care providers should not allow themselves to be influenced by strong personal bonds between their patients and their family members.	3.633	1.0618	4.378	1.1322	0.000*
I do not enjoy reading non-medical literature or the arts.	3.117	1.1100	4.218	1.2197	0.000*
I believe that empathy is an important factor in patients' treatment.	4.394	1.0754	5.295	1.2913	0.000*
Total empathy score	76.217	5.4283	91.526	7.3585	0.000*

**Table 2:** Comparison of self-reported clinical scores and clinical exam scorebetween NSS and Non NSS volunteers.

Items	Non NSS volunteers		NSS volunteers		P value
	Mean	SD	Mean	SD	
History recording and clinical examination	3.517	.6471	4.179	.4470	0.000*
Radiography	3.439	.7487	3.974	.4945	0.000*
Case diagnosis	3.267	.6395	3.763	.5578	0.000*
Treatment planning	3.322	.7064	3.833	.6100	0.000*
Oral hygiene instructions	4.128	.5182	4.231	.5186	0.070
Oral prophylaxis	4.106	.5932	4.449	.5483	0.000*
Fluoride application	3.211	1.0675	4.282	.5054	0.000*
Pit and Fissure sealants	3.594	.7959	4.468	.5497	0.000*
Preventive resin restorations	3.061	1.0526	4.359	.5897	0.000*
Sub gingival scaling	3.122	.8499	3.494	.8688	0.000*
Root planning	2.378	.9164	2.545	1.1379	0.137
Minor periodontal surgeries	2.172	.8511	2.532	1.1440	0.001*
Rubber dam placement	2.372	.9574	3.724	.6679	0.000*
Amalgam/GIC/Composite restorations	3.606	1.1061	4.346	.4773	0.000*
Pulp capping procedures	3.150	.9658	4.103	.6136	0.000*
Anterior teeth endodontics	3.072	1.0625	4.026	.5559	0.000*
Administration of all forms of local anesthesia	3.550	.9110	3.994	.6574	0.000*
Administration of intra muscular and venous injections	2.761	1.0590	2.897	.9717	0.222
Simple extractions	4.033	.8516	4.288	.5081	0.001*
Trans-alveolar extractions	2.567	1.0523	2.699	.9667	0.234
Frenectomy	2.200	.9825	2.538	1.2307	0.005*
Dento alveolar procedures	2.356	.9373	2.571	1.1194	0.056
Simple impaction	2.344	.9295	2.679	1.3198	0.007*
Biopsy	2.271	.9856	2.487	1.2048	0.073
Simple suturing	3.133	1.1005	3.897	.6737	0.000*
Perform basic cardiac life support	2.778	.9249	3.359	.7180	0.000*
Prescription of drugs, pre-operative/prophylactic/therapeutic requirements	3.144	.7989	3.615	.6764	0.000*
Manage common complications that arise during/after minor oral surgery	2.933	.9665	3.282	.6890	0.000*
Manage medical emergencies in the dental office	2.828	.7898	3.173	.7965	0.000*
Removable partial dentures	3.567	.6525	3.795	.6497	0.001*
Complete denture construction	3.256	.7848	3.994	.5388	0.000*
Fixed partial dentures	2.639	.8829	3.109	.9475	0.000*
Treatment of pediatric patients	3.389	.7429	3.462	.6464	0.343
Simple orthodontic appliance therapy	2.856	1.0090	2.853	1.1234	0.980
Appointment setting and communication with the patient	3.800	.7578	4.103	.6444	0.000*
Total Self-confidencescore	3.082	.437	3.574	.342	0.000*
Clinical exam score	445.03	34.940	501.15	53.970	0.000*

## Data Availability

The datasets used and/or analyzed during the current study are available from the corresponding author.
